# Clinical outcomes of patients with lymphoid blastic phase of chronic myeloid leukemia treated with CAR T-cell therapy

**DOI:** 10.1038/s41408-024-01020-y

**Published:** 2024-03-06

**Authors:** Yujie Liu, Yuqing Tu, Jinyan Xiao, Yifan Shen, Biqi Zhou, Qiannan Yang, Lei Yu, Lijuan Qi, Jia Chen, Tianhui Liu, Depei Wu, Yang Xu

**Affiliations:** 1 Jiangsu Institute of Hematology, National Clinical Research Center for Hematologic Diseases, The First Affiliated Hospital of Soochow University, Suzhou, P. R. China; 2 Institute of Blood and Marrow Transplantation, Collaborative Innovation Center of Hematology, Soochow University, Suzhou, P. R. China; 3 Shanghai Unicar Therapy Bio-Medicine Technology Co. Ltd, Shanghai, China

**Keywords:** Acute lymphocytic leukaemia, Chronic myeloid leukaemia, Cancer immunotherapy

**TO THE EIDTOR:**

Chronic myeloid leukemia (CML) is a triphasic myeloproliferative disorder that accounts for 15–20% of adult leukemias [1]. The cytogenetic hallmark is a reciprocal translocation between chromosomes 9 and 22, resulting in *BCR::ABL1* fusion gene. Lymphoid blast crisis (LBC) is one of the most dreaded complications of CML, with the B-cell lineage being more common [2]. Although the incidence of CML-LBC has decreased markedly in the tyrosine kinase inhibitor (TKI) era, responses to TKIs in CML-LBC are infrequent and of short-lived [3]. Chimeric antigen receptor T (CAR T) is an effective therapy for refractory/relapsed (R/R) B acute lymphoblastic leukemia (ALL) patients [4–6], but the therapeutic response and long-term prognosis of CML-LBC patients are unclear. We retrospectively collected the clinical data from 13 CML-LBC patients who received CAR T treatment at our center from February 2017 to March 2023 and compared their outcomes with those of 121 Philadelphia chromosome-positive (Ph + ) ALL patients who underwent CAR T treatment at our center during the same period (Figs. S2B and S3A). All patients were diagnosed and differentially diagnosed by past medical history, morphology, immunology, cytogenetics and molecular biology. This study was approved by the Institutional Review Board of our hospital.

The clinical timeline of CML-LBC patients is shown in Fig. 1A. Baseline characteristics of the two groups are summarized in Table S1 and the use of TKIs of these patients are shown in Fig. S1. The CAR T therapy was given for both overt relapse and MRD-positive patients. Before CAR T-cell treatment, 7.7% (1/13) CML-LBC patients and 9.9% (12/121) Ph+ ALL patients had primary refractory disease, 38.5% (5/13) CML-LBC patients and 16.5% (20/121) Ph+ ALL patients experienced the first relapse, 7.7% (1/13) CML-LBC patients and 9.9% (12/121) Ph+ ALL patients experienced a second or more relapse, and 46.2% (6/13) CML-LBC patients and 63.6% (77/121) Ph+ ALL patients experienced MFC MRD positive. Among all MFC MRD-positive patientis, BCR::ABL1 transcripts were detected by PCR in some of them, but in a few patients the test results were negative (Fig. S3B, Table S6). Notably, patients with CML-LBC carried a higher proportion of ABL kinase domain mutations (46.2%, 6/13 vs. 17.4%, 21/121; *P* = 0.036) (Table S1). The ABL kinase domain mutations and other gene mutations in the cohort are summarized in Fig. S2A. Response to treatment was judged by morphological, cytological or molecular level. For refractory and relapse patients, response to treatment means reaching CR. For MRD-positive patients, response to treatment means persistent morphological CR or reaching CMR (Supplemental data 2). The treatment response rate (61.5%, 8/13 vs. 90.8%, 108/119; *P* = 0.009) of CML-LBC patients was significantly lower than that of Ph+ ALL patients after CAR T-cell infusion, as was the MRD-negative CR rate (23.1%, 3/13 vs. 74.8%, 89/119; *P* < *0.001*) (Fig. 1B–C). Furthermore, the MRD-negative CR rate of R/R (14.3%, 1/7 vs. 57.1%, 24/42; *P* = 0.049) and MRD positive (33.3%, 2/6 vs. 84.4%, 65/77; *P* = 0.011) patients with CML-LBC were also significantly lower than those in Ph+ ALL patients (Fig. S2C). Univariate analyses revealed that overt relapse status, BM blasts ≥ 10%, ABL kinase domain mutation and CML-LBC were significantly associated with an unfavorable therapeutic response (Table S2 and S3). Through multivariate logistic regression analyses of CR and MRD-negative CR, CML-LBC were also an independent risk factor for therapeutic response (Table 1). Moreover, ABL kinase region mutation remained a significant independent predictor of poor treatment response (Table 1).

**Fig. 1:**
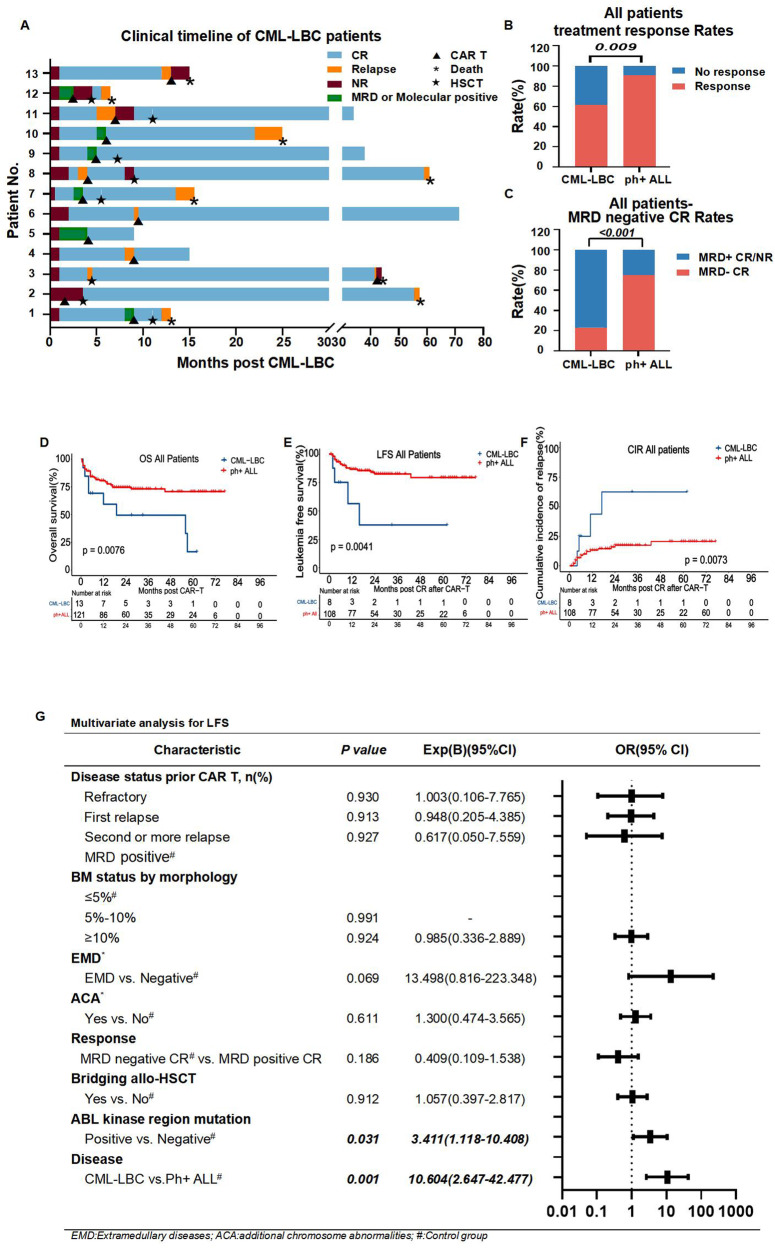
Clinical timeline of CML-LBC patients, treatment response rates, MRD negative CR rates, OS, LFS, CIR and Multivariate analysis for LFS. **A** The clinical timeline of 13 patients with CML-LBC; **B** The treatment response rate of CML-LBC and Ph+ ALL patients; **C** The MRD-negative complete remission rate of CML-LBC and Ph+ ALL patients; **D** Overall survival of CML-LBC and Ph+ ALL patients; **E** Leukemia free survival of CML-LBC and Ph+ ALL patients; **F** Cumulative incidence of relapse of CML-LBC and Ph+ ALL patients; **G** Cox regression multivariable analysis for LFS.

**Table 1:** Multivariate analysis for treatment response post-CAR T.

Characteristic	Response		MRD-negative CR	
	OR (95%CI)	*P* value	OR (95%CI)	*P* value
Disease status prior CAR T, *n* (%)				
Refractory	0.037 (0.001–2.104)	0.111	2.865 (0.097–84.997)	0.543
First relapse	0.274 (0.004–19.774)	0.553	5.141 (0.186–141.972)	0.334
Second or more relapse	0.037 (0.000–2.930)	0.140	2.693 (0.076–95.294)	0.586
MRD Positive^a^				
Prior allo-HSCT				
Yes vs. No^a^	0.277 (0.026–2.944)	0.287	0.966 (0.230–4.064)	0.963
EMD				
EMD vs. Negative^a^	0.159 (0.012–2.153)	0.166	0.232 (0.031–1.711)	0.152
BM status by morphology				
≤5%^a^				
5–10%	–	0.999	0.195 (0.003–14.221)	0.455
≥10%	0.547 (0.016–18.153)	0.735	0.098 (0.004–2.326)	0.151
ACA				
Yes vs. No^a^	1.807 (0.332–9.826)	0.494	1.202 (0.442–3.270)	0.719
Target				
Single CD19^a^ vs. CD19/CD22	0.617 (0.118–3.230)	0.568	0.405 (0.151–1.085)	0.072
ABL kinase region mutation				
Positive vs. Negative^a^	***0.179 (0.037***–***0.857)***	***0.031***	0.401 (0.131–1.225)	0.109
Disease				
CML-LBC vs. Ph+ ALL^a^	***0.101 (0.010***–***0.966)***	***0.047***	***0.103 (0.022***–***0.483)***	***0.004***

*EMD* Extramedullary diseases, *ACA* additional chromosome abnormalities; ^a^Control group.

Bold values indicates statistically significant *P* values less than 0.05.

All adverse events and organ toxicity associated with CAR T-cell therapy were graded as shown in Table S4. CRS occurred in 44.0% (59/134) patients, and severe CRS (grade 3-4) occurred in 7.7% (1/13) of CML-LBC patients and 8.3% (10/121) of Ph+ ALL patients (*P* = 1.000). There was no significant difference in the incidence of adverse events and organ toxicity events between the two groups. One hundred and twenty-six patients had their levels of serum cytokines monitored regularly from the day of CAR T-cells infusion, and consistent results were detected in the two groups (Fig. S2D). Besides, there was no statistically significant difference between the two groups with regard to CAR T-cell copy numbers within one month after infusion (Fig. S2E). However, in all patients with active disease at baseline, CML-LBC patients (*n* = 7) had lower CAR T-cells copies peak (*P* = 0.003) than Ph+ ALL patients (*n* = 44). Moreover, there was no statistically significant difference in CAR T-cells expansion in MRD-positive patients between the two groups (Fig. S2F).

The median follow-up time of all patients was 30 months (range 0.5–77 months). The 2-year prognosis of CML-LBC patients was worse than that of Ph+ ALL patients (overall survival (OS): 49.5% vs. 74.5%, *P* = 0.0076; leukemia-free survival (LFS): 37.5% vs. 82.6%, *P* = 0.0041; cumulative incidence of relapse (CIR): 83.9% vs. 37.5%, *P* = 0.0073) (Fig. 1D–F). In addition, among all patients bridging allo-HSCT (CML-LBC, *n* = 3；Ph+ ALL, *n* = 47), 66.7% (2/3) CML-LBC patients and 19.1% (9/47) Ph+ ALL patients experienced relapse. Univariable analyses were performed to identify baseline and therapy-related factors associated with improved LFS in patients who achieved CR and could be included in subsequent multivariable analyses (Table S6). In addition to the worse LFS in CML-LBC patients described above (Fig. 1E), patients with ABL kinase domain mutations showed a significantly worse LFS than those without ABL kinase domain mutations (2-year LFS: 49.9% vs. 84.5%, *P* = 0.0024) (Fig. S2G). Cox regression multivariable model also showed that patients carrying ABL kinase region mutations (HR: 3.411, 95% CI: 1.118–10.408; *P* = 0.031) and patients with CML-LBC (HR: 10.604, 95% CI: 2.647–42.477; *P* = 0.001) were independent risk factors for LFS (Fig. 1G).

Recently, TKIs have revolutionized the treatment of CML, clinical outcomes remain suboptimal for LBC [7]. While a small number of case reports also elaborated their experience with CAR T treatment for CML-LBC patients [8, 9], long-term follow-up and comparative analyses with other patients are still lacking, but are needed to determine the true efficacy of CAR T therapy in CML-LBC patients. In this study, we compared the efficacy and safety of CAR T-cell therapy in patients with CML-LBC and Ph+ ALL. We found worse treatment response and prognoses in CML-LBC patients and there was no evidence of difference in toxicity. The higher frequency of ABL kinase domain mutations and poor CAR T cells expansion may account for worse outcomes in CML-LBC patients. Hitherto, more than 100 mutations have been reported to be related to various degrees of resistance to TKIs [10]. Among them, T315I and P-loop mutations are associated with the worst clinical outcome and patient’s rapid entrance into the blast phase [11, 12]. Moreover, reduced CAR T-cell persistence, T-cell exhaustion and tumor intrinsic factors have been identified as mechanisms of CAR T resistance. Several reports suggested that a regimen of CAR T cells combined with BTKs is well tolerated in R/R CLL patients, with low CRS severity and high response rates [13, 14]. Hence, it is of paramount importance to select TKIs and further explore the efficacy and safety of TKIs combined with CAR T-cell therapy in CML-LBC patients. In addition, In cases of de novo Ph+ ALL, the neoplasm arises from a committed lymphoid progenitor cell, which is the rationale for currently approved CAR T-cell therapies. However, it is well known that at least two groups of clones exist in CML-LBC, one in the chronic phase and the other in the blast phase [15]. CAR T-cell therapy is effective only against cancerous B lineage cells but fails to eliminate target-free blasts derived from CML stem cells. We propose that future studies may find potential in exploring the use of a bispecific or compound CAR that can target both myeloid and lymphoid precursor leukemia cells. We acknowledge that case-size limitations preclude more detailed subgroup analyses, and real-world clinical trials are needed to more accurately investigate the efficacy of CAR T therapy in CML-LBC patients.

In conclusion, we retrospectively compared the outcomes of CAR T therapy in patients with CML-LBC and Ph+ ALL. CML-LBC was an independent risk factor of treatment response and survival profiles. Additionally, we observed a higher proportion of ABL kinase domain mutations in CML-LBC patients, possibly responsible for unfavorable outcomes.

### Supplementary information

supplemental data

## References

[1] Siegel RL, Miller KD, Jemal A (2017). Cancer Statistics, 2017. CA Cancer J Clin.

[2] Swerdlow SH, Campo E, Pileri SA, Harris NL, Stein H, Siebert R (2016). The 2016 revision of the World Health Organization classification of lymphoid neoplasms. Blood.

[3] Hehlmann R (2012). How I treat CML blast crisis. Blood.

[4] Lim WA, June CH (2017). The Principles of Engineering Immune Cells to Treat Cancer. Cell.

[5] Chen L, Gong W-J, Li M-H, Zhou H-X, Xu M-Z, Qian C-S (2023). Anti-CD19 CAR T-Cell consolidation therapy combined with CD19+ feeding T cells and TKI for Ph+ acute lymphoblastic leukemia. Blood Adv.

[6] Hua J, Qian W, Wu X, Zhou L, Yu L, Chen S (2020). Sequential Infusion of Anti-CD22 and Anti-CD19 Chimeric Antigen Receptor T Cells for a Pediatric Ph-Like B-ALL Patient That Relapsed After CART-Cell and Haplo-HSCT Therapy: A Case Report and Review of Literature. Onco Targets Ther.

[7] Giles FJ, Kantarjian HM, le Coutre PD, Baccarani M, Mahon FX, Blakesley RE (2012). Nilotinib is effective in imatinib-resistant or -intolerant patients with chronic myeloid leukemia in blastic phase. Leukemia.

[8] Liu F, Sha S, Ma G, Su Y, Xiong Y, He G (2020). Treatment of CML-transformed B Cell Acute Lymphoblastic Leukemia (B-ALL) in Adults with Anti-CD19 Chimeric Antigen Receptor T Cell (CAR T) Therapy. Stem Cell Rev Rep..

[9] Zhou L, Shi H, Shi W, Yang L, Zhang Y, Xu M (2019). Durable Molecular Remission in a Lymphoid BP-CML Patient Harboring T315I Mutation Treated with Anti-CD19 CAR-T Therapy. Onco Targets Ther.

[10] Soverini S, Hochhaus A, Nicolini FE, Gruber F, Lange T, Saglio G (2011). BCR-ABL kinase domain mutation analysis in chronic myeloid leukemia patients treated with tyrosine kinase inhibitors: recommendations from an expert panel on behalf of European LeukemiaNet. Blood.

[11] O’Hare T, Eide CA, Deininger MWN (2007). Bcr-Abl kinase domain mutations, drug resistance, and the road to a cure for chronic myeloid leukemia. Blood.

[12] Bengió RM, Riva ME, Moiraghi B, Lanari E, Milone J, Ventriglia V (2011). Clinical outcome of chronic myeloid leukemia imatinib-resistant patients: do BCR-ABL kinase domain mutations affect patient survival? First multicenter Argentinean study. Leuk Lymphoma.

[13] Gauthier J, Hirayama AV, Purushe J, Hay KA, Lymp J, Li DH (2020). Feasibility and efficacy of CD19-targeted CAR T cells with concurrent ibrutinib for CLL after ibrutinib failure. Blood.

[14] Kamdar M (2023). Embracing chimeric antigen receptors for relapsed chronic lymphocytic leukaemia. Lancet.

[15] Jamieson CHM, Ailles LE, Dylla SJ, Muijtjens M, Jones C, Zehnder JL (2004). Granulocyte-macrophage progenitors as candidate leukemic stem cells in blast-crisis CML. N. Engl J Med.

