# Supplementary material for: Clinical outcomes of patients with lymphoid blastic phase of chronic myeloid leukemia treated with CAR T-cell therapy
Source: Blood Cancer J. 2024 Mar 6;14(1):39. doi: 10.1038/s41408-024-01020-y (PMC10917735; doi:10.1038/s41408-024-01020-y)
Supplement: Supplementary file 1 — supplemental data [file 41408_2024_1020_MOESM1_ESM.pdf]

## Figure S1

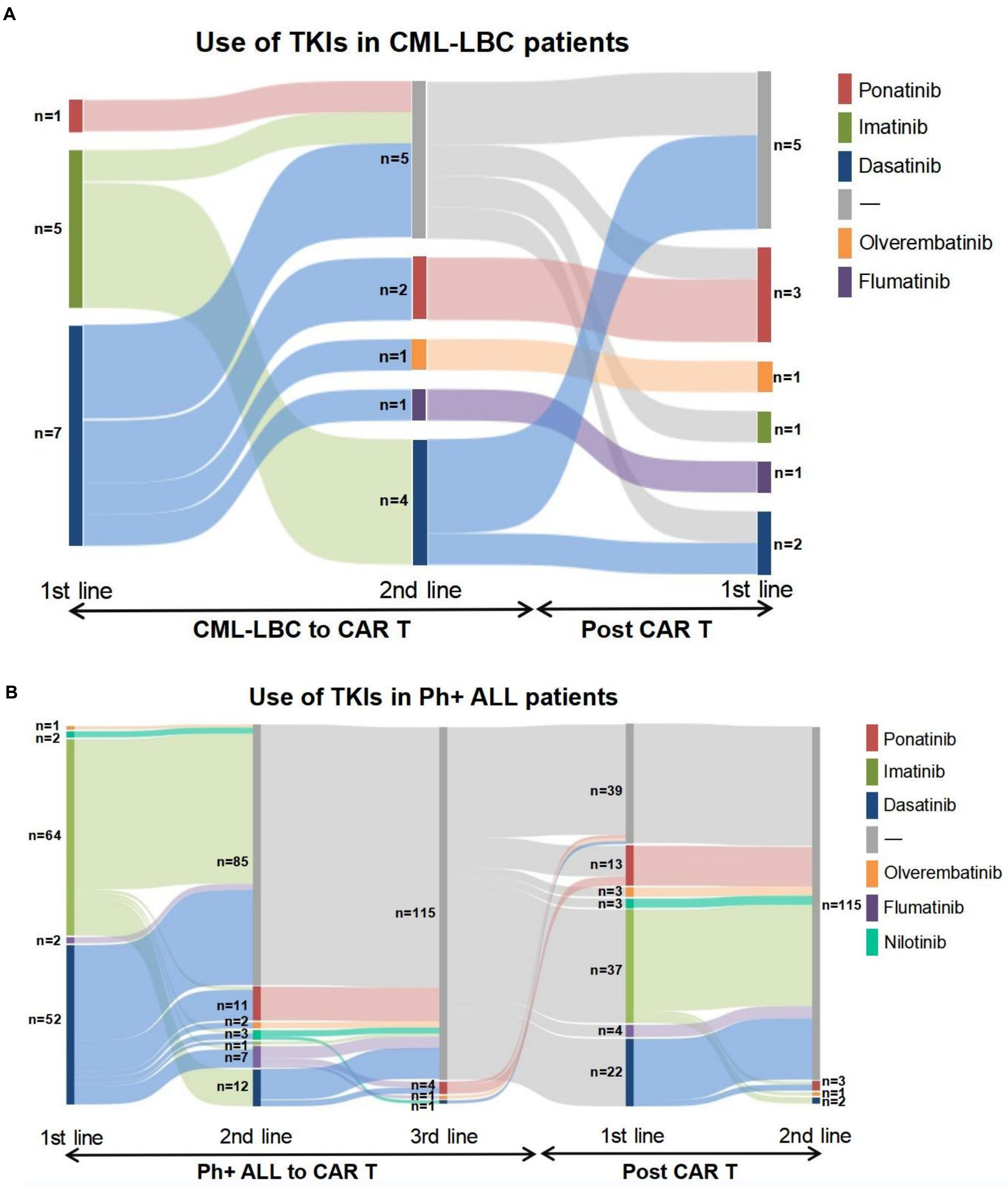


**Figure legends:**

**Figure S1:** (A) Use of TKIs in CML-LBC patients; (B) Use of TKIs in Ph+ ALL patients.

# Figure S2

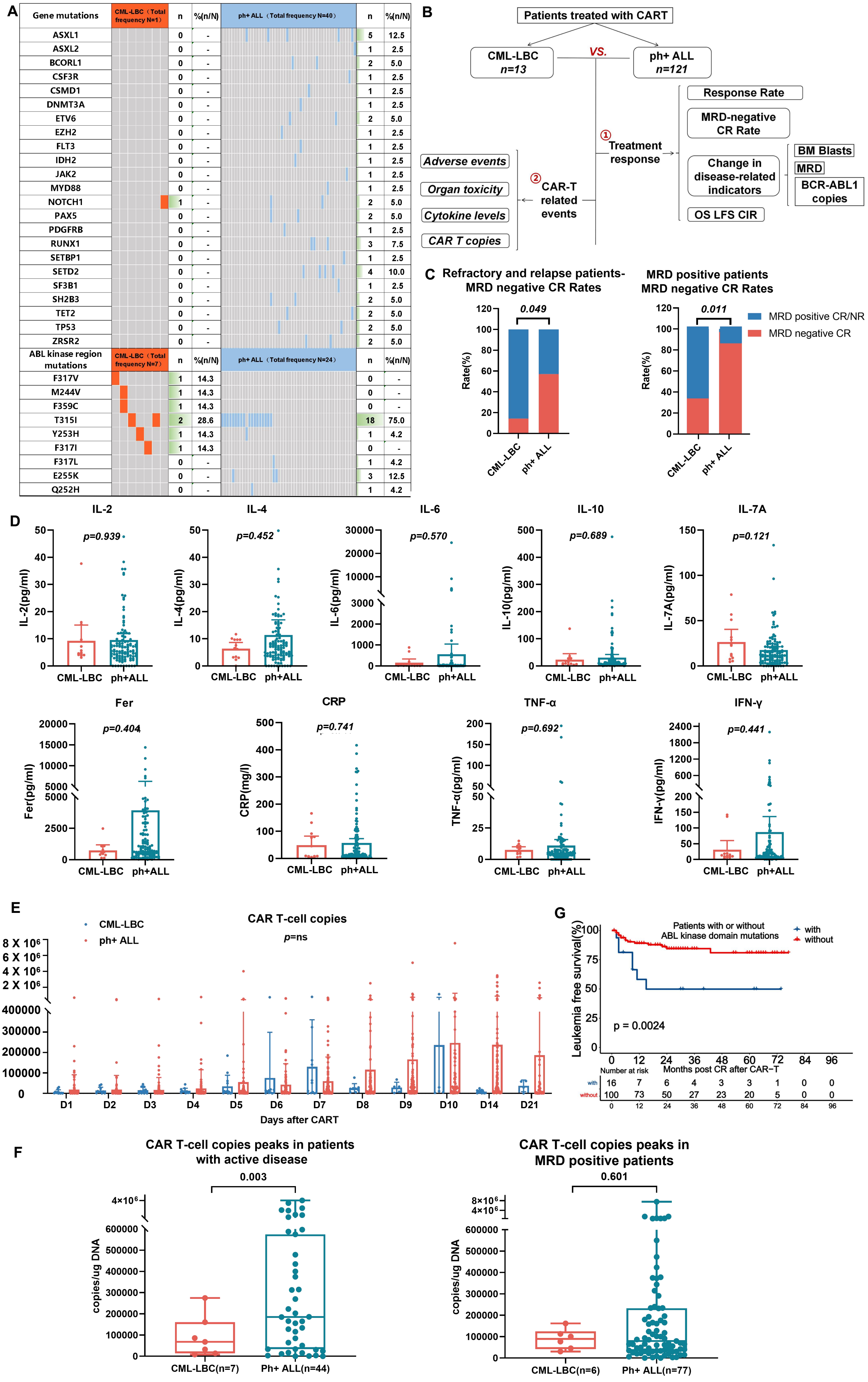


**Figure legends:**

**Figure S2:** (A) The ABL kinase domain mutations and other gene mutations in the cohort; (B) Research flow chart; (C) MRD-negative complete remission rate of refractory/relpase and MRD positive patients among the two groups; (D) The levels of serum cytokines; (E) CAR T-cell copy numbers within one month after infusion; (F) CAR T-cell copies peaks in patients with active disease and in MRD positive patients; (G) Leukemia free survival of patients with or without ABL kinase domain mutations.

# Figure S3

A

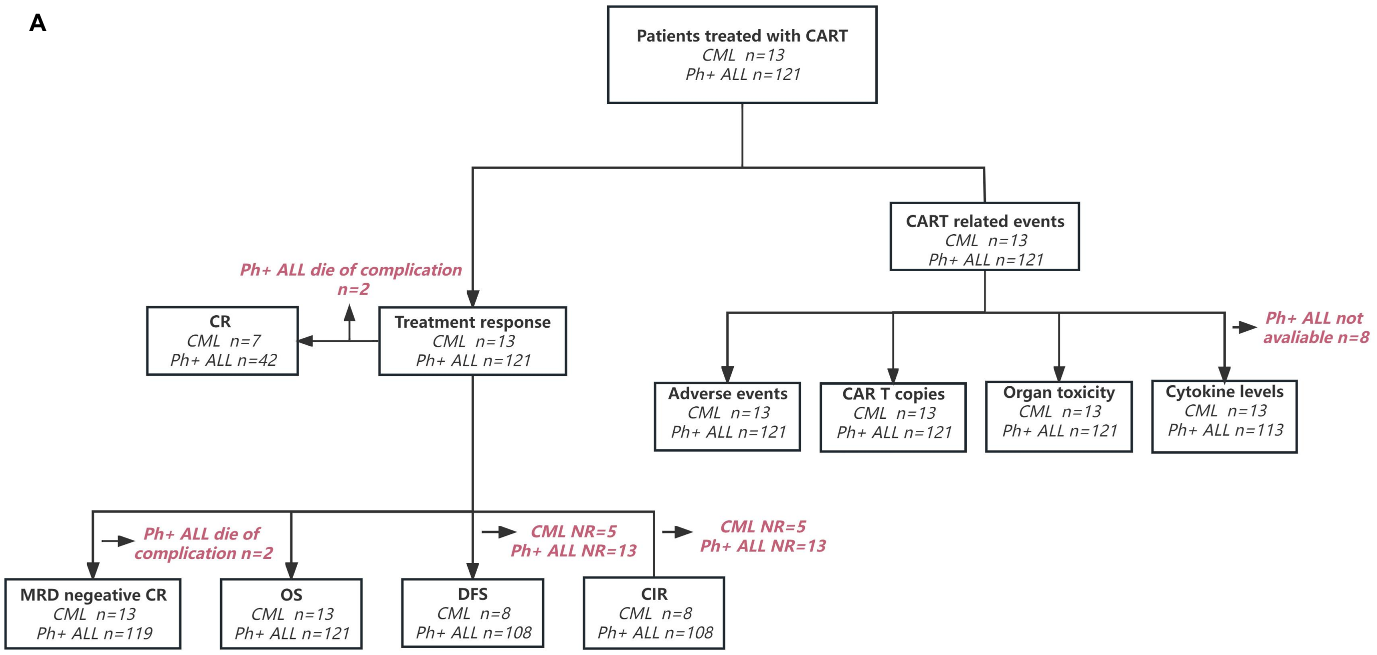


B

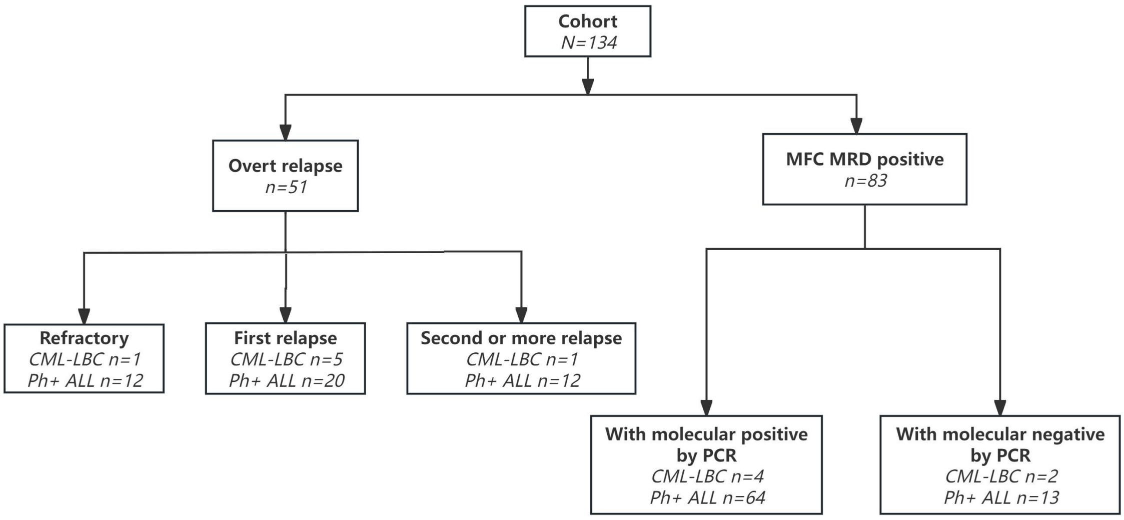


## Figure legends:

**Figure S3A:** Research flow chart;

**Figure S3B:** Disease status prior CAR T therapy in the cohort (N=134);

Table S1:Baseline characteristics of patients

| Characteristic | CML-LBC (n=13) | Ph+ ALL (n=121) | *P value* |
|---|---|---|---|
| **Age, yr, median (range)** | 32(17-53) | 42(11-68) | 0.074 |
| **Sex, n(%)** | | | 0.354 |
| Male | 7(53.8) | 49(40.5) | |
| Female | 6(46.2) | 72(59.5) | |
| **Disease status prior CAR T, n(%)** | | | |
| Refractory | 1(7.7) | 12(9.9) | 1.000 |
| First relapse | 5(38.5) | 20(16.5) | 0.120 |
| Second or more relapse | 1(7.7) | 12(9.9) | 1.000 |
| MRD positive | 6(46.2) | 77(63.6) | 0.351 |
| **Fusion gene type,n(%)** | | | 0.734 |
| P190 | 0(0.0) | 113(93.4) | |
| P210 | 13(100.0) | 8(6.6) | |
| P230 | 0(0.0) | 0(0.0) | |
| **Course of prior therapy** | | | 0.189 |
| ≤3 | 11(84.6) | 75(62.0) | |
| ≥4 | 2(15.4) | 46(38.0) | |
| **Course of prior TKIs, median (range)** | 2(1-2) | 1(1-3) | 0.108 |
| **Prior allo-HSCT** | | | 1.000 |
| Yes | 1(7.7) | 14(11.6) | |
| No | 12(92.3) | 107(88.4) | |
| **EMD*** | | | 0.615 |
| EMD | 2(15.4) | 10(8.3) | |
| Negative | 11(84.6) | 111(91.7) | |
| **BM status by morphology** | | | 0.483 |
| ≤5% | 7(53.8) | 80(66.1) | |
| 5%-10% | 0(0.0) | 2(1.65) | |
| ≥10% | 6(46.2) | 39(32.2) | |
| **ACA*** | | | 1.000 |
| Yes | 4(30.8) | 41(33.9) | |
| No | 9(69.2) | 80(66.1) | |
| **Target** | | | 0.420 |
| Single CD19 | 8(61.5) | 92(76.0) | |
| CD19/CD22 | 5(38.5) | 29(24.0) | |
| **ABL kinase region mutation** | | | ***0.036*** |
| Positive | 6(46.2) | 21(17.4) | |
| Negative | 7(53.8) | 100(82.6) | |

*EMD:Extramedullary diseases; ACA:additional chromosome abnormalities;*

**Table S2:Univariate analysis for Response rate post-CAR T**

| Characteristic | Response | *P value* | Exp(B)(95%CI) |
|---|---|---|---|
| **Age,yr** | | | |
| ≤14 | 2/3(66.7) | 0.347 | 0.276(0.022-3.400) |
| 15-39 | 56/63(88.9) | 0.326 | 0.250(0.020-3.125) |
| ≥40# | 58/66(87.9) | | |
| **Sex** | | | |
| Male# | 48/56(85.7) | | |
| Female | 68/76(89.5) | 0.513 | 1.417(0.479-4.038) |
| **Disease status prior CAR T, n(%)** | | | |
| Refractory | 7/12(58.3) | ***0.000*** | ***0.017(0.002-0.167)*** |
| First relapse | 20/25(80.0) | ***0.002*** | ***0.049(0.005-0.441)*** |
| Second or more relapse | 7/12(58.3) | ***0.000*** | ***0.017(0.002-0.167)*** |
| MRD positive# | 82/83(98.8) | | |
| **Course of prior therapy** | | | |
| ≤3# | 76/85(89.4) | | |
| ≥4 | 40/47(85.1) | 0.468 | 0.677(0.235-1.952) |
| **Prior allo-HSCT** | | | |
| Yes | 12/15(80.0) | 0.567 | 0.500(0.124-2.008) |
| No# | 104/117(88.9) | | |
| **EMD*** | | | |
| EMD | 7/11(63.6) | ***0.037*** | ***0.193(0.049-0.755)*** |
| Negative# | 109/121(90.1) | | |
| **BM status by morphology** | | | |
| ≤5%# | 85/87(97.7) | | |
| 5%-10% | 2/2(100.0) | 1.000 | - |
| ≥10% | 29/43(67.4) | ***0.000*** | ***0.049(0.010-0.227)*** |
| **ACA*** | | | |
| Yes | 38/44(86.4) | 0.706 | 0.812(0.275-2.400) |
| No# | 78/88(88.6) | | |
| **Target** | | | |
| Single CD19# | 89/99(89.9) | | |
| CD19/CD22 | 27/33(81.8) | 0.356 | 0.506(0.168-1.519) |
| **ABL kinase region mutation** | | | |
| Positive | 16/26(61.5) | ***0.000*** | ***0.096(0.031-0.301)*** |
| Negative# | 100/106(94.3) | | |
| **Disease** | | | |
| CML-LBC | 8/13(61.5) | ***0.009*** | ***0.163(0.045-0.585)*** |
| Ph+ ALL# | 108/119(90.8) | | |

*EMD:Extramedullary diseases; ACA:additional chromosome abnormalities; #:Control group*

**Table S3:Univariate analysis for MRD-negative CR rate post-CAR T**

| Characteristic | CR rate | *P value* | Exp(B)(95%CI) |
|---|---|---|---|
| **Age,yr** | | | |
| ≤14 | 2/3(66.7) | 1.000 | 0.750(0.064-8.786) |
| 15-39 | 42/63(66.7) | 0.454 | 0.750(0.353-1.593) |
| ≥40# | 48/66(72.7) | | |
| **Sex** | | | |
| Male# | 36/56(64.3) | | |
| Female | 56/76(73.7) | 0.246 | 1.556(0.736-3.286) |
| **Disease status prior CAR T, n(%)** | | | |
| Refractory | 6/12(50.0) | ***0.046*** | ***0.239(0.068-0.839)*** |
| First relapse | 14/25(56.0) | ***0.012*** | ***0.304(0.116-0.794)*** |
| Second or more relapse | 5/12(41.7) | ***0.010*** | ***0.171(0.048-0.608)*** |
| MRD positive# | 67/83(80.7) | | |
| **Course of prior therapy** | | | |
| ≤3# | 62/85(72.9) | | |
| ≥4 | 30/47(63.8) | 0.275 | 0.655(0.305-1.405) |
| **Prior allo-HSCT** | | | |
| Yes | 10/15(66.7) | 1.000 | 0.854(0.272-2.680) |
| No# | 82/117(70.1) | | |
| **EMD*** | | | |
| EMD | 5/11(45.5) | 0.138 | 0.326(0.093-1.138) |
| Negative# | 87/121(71.9) | | |
| **BM status by morphology** | | | |
| ≤5%# | 70/87(80.5) | | |
| 5%-10% | 1/2(50.0) | 0.356 | 0.243(0.014-4.083) |
| ≥10% | 21/43(48.8) | ***0.000*** | ***0.232(0.104-0.515)*** |
| **ACA*** | | | |
| Yes | 31/44(70.5) | 0.893 | 1.055(0.479-2.326) |
| No# | 61/88(69.3) | | |
| **Target** | | | |
| Single CD19# | 74/99(74.7) | | |
| CD19/CD22 | 18/33(54.5) | ***0.029*** | ***0.405(0.178-0.922)*** |
| **BCR-ABL kinase region mutation** | | | |
| Positive | 11/26(42.3) | ***0.001*** | ***0.226(0.092-0.556)*** |
| Negative# | 81/106(76.4) | | |
| **Disease** | | | |
| CML-LBC | 3/13(23.1) | ***0.000*** | ***0.101(0.026-0.392)*** |
| Ph+ ALL# | 89/119(74.8) | | |

*EMD:Extramedullary diseases; ACA:additional chromosome abnormalities; #:Control group*

**Table S4:AEs after CAR T-cell infusion**

| AEs | CML-LBC(n=13) | Ph+ ALL(n=121) | *P value* |
|---|---|---|---|
| **Fatigure** | | | 0.210 |
| Grade 0 | 5(38.5) | 74(61.2) | |
| Grade 1-2 | 8(61.5) | 44(36.4) | |
| Grade 3-4 | 0(0.0) | 3(2.5) | |
| **Nausea+anorexia** | | | 0.108 |
| Grade 0 | 6(46.2) | 88(72.7) | |
| Grade 1-2 | 7(53.8) | 31(25.6) | |
| Grade 3-4 | 0(0.0) | 2(1.7) | |
| **Diarrhea+constipation** | | | 0.394 |
| Grade 0 | 10(76.9) | 108(89.3) | |
| Grade 1-2 | 3(23.1) | 13(10.7) | |
| Grade 3-4 | 0(0.0) | 0(0.0) | |
| **Infection** | | | 0.444 |
| Grade 0 | 8(61.5) | 90(74.4) | |
| Grade 1-2 | 0(0.0) | 2(1.7) | |
| Grade 3-4 | 5(38.5) | 29(24.0) | |
| **Fever** | | | 0.064 |
| Grade 0 | 4(30.8) | 67(55.4) | |
| Grade 1-2 | 9(69.2) | 41(33.9) | |
| Grade 3-4 | 0(0.0) | 13(10.7) | |
| **Hypotension** | | | 1.000 |
| Grade 0 | 12(92.3) | 106(87.6) | |
| Grade 1-2 | 0(0.0) | 5(4.1) | |
| Grade 3-4 | 1(7.7) | 10(8.3) | |
| **Hypoxia** | | | 0.613 |
| Grade 0 | 12(92.3) | 113(93.4) | |
| Grade 1-2 | 0(0.0) | 1(0.8) | |
| Grade 3-4 | 1(7.7) | 7(5.8) | |
| **CRS** | | | 0.101 |
| Grade 0 | 4(30.8) | 71(58.7) | |
| Grade 1-2 | 8(61.5) | 40(33.1) | |
| Grade 3-4 | 1(7.7) | 10(8.3) | |
| **Neurotoxicity** | 1(7.7) | 0(0.0) | 0.088 |
| **Hematologic AEsNeutropenia** | | | 0.604 |
| Grade 0 | 5(38.5) | 37(30.6) | |
| Grade 1-2 | 4(30.8) | 29(24.0) | |
| Grade 3-4 | 4(30.8) | 55(45.5) | |
| **Thrombocytopenia** | | | 0.875 |
| Grade 0 | 4(30.8) | 36(29.8) | |
| Grade 1-2 | 6(46.2) | 47(38.8) | |
| Grade 3-4 | 3(23.1) | 38(31.4) | |
| **Hypoglobinemia** | | | 0.907 |
| Grade 0 | 1(7.7) | 9(7.4) | |
| Grade 1-2 | 7(53.8) | 59(48.8) | |
| Grade 3-4 | 5(38.5) | 53(43.8) | |
| **Laboratory abnormalitiesALT/AST*elevation** | | | 0.392 |
| Grade 0 | 9(69.2) | 99(81.8) | |
| Grade 1-2 | 4(30.8) | 20(16.5) | |
| Grade 3-4 | 0(0.0) | 2(1.7) | |
| **Blood bilirubin elevation** | | | 0.463 |
| Grade 0 | 8(61.5) | 91(75.2) | |
| Grade 1-2 | 5(38.5) | 30(24.8) | |
| Grade 3-4 | 0(0.0) | 0(0.0) | |
| **Electrolyte disturbance** | | | 0.410 |
| Grade 0 | 9(69.2) | 78(64.5) | |
| Grade 1-2 | 3(23.1) | 40(33.1) | |
| Grade 3-4 | 1(7.7) | 3(2.5) | |
| **NEsInsomnia** | | | 0.382 |
| Grade 0 | 8(61.5) | 91(75.2) | |
| Grade 1-2 | 5(38.5) | 29(24.0) | |
| Grade 3-4 | 0(0.0) | 1(0.8) | |
| **Headache/dizziness** | | | 0.444 |
| Grade 0 | 10(76.9) | 105(86.8) | |
| Grade 1-2 | 3(23.1) | 15(12.4) | |
| Grade 3-4 | 0(0.0) | 1(0.8) | |

**ALT: alanine aminotransferase; AST: aspartate aminotransferase.*

**Table S5: Univariate analysis for LFS**

| Characteristic | 2-year LFS | *P value* | Exp(B)(95%CI) |
|---|---|---|---|
| **Age,yr** | | | |
| ≤14 | 100.0% | 0.485 | - |
| 15-39 | 80.7.% | 0.767 | 1.137(0.483-2.675) |
| ≥40# | 78.1% | | |
| **Sex** | | | |
| Male# vs. Female | 86.0% vs. 75.8% | 0.448 | 1.416(0.594-3.373) |
| **Disease status prior CAR T, n(%)** | | | |
| Refractory | 71.4% | 0.384 | 1.899(0.278-12.960) |
| First relapse | 82.0% | 0.969 | 1.025(0.292-3.603) |
| Second or more relapse | 40.0% | 0.351 | 1.986(0.280-14.070) |
| MRD positive# | 82.2% | | |
| **Course of prior therapy** | | | |
| ≤3# vs. ≥4 | 78.6% vs. 82.1% | 0.459 | 0.688(0.373-0.731) |
| **Prior allo-HSCT** | | | |
| Yes vs. No# | 88.9% vs. 78.7% | 0.380 | 0.422(0.105-1.691) |
| **EMD*** | | | |
| EMD vs. Negative# | 57.1% vs. 82.3% | 0.258 | 2.250(0.284-17.830) |
| **BM status by morphology** | | | |
| ≤5%# | 82.5% | | |
| 5%-10% | 100.0% | 0.536 | - |
| ≥10% | 70.0% | 0.272 | 1.648(0.599-4.534) |
| **ACA*** | | | |
| Yes vs. No# | 76.3% vs. 81.6% | 0.379 | 1.467(0.594-3.623) |
| **Response** | | | |
| MRD-negative-CR# vs. MRD-positive-CR | 79.5% vs. 82.5% | 0.669 | 0.789(0.283-2.210) |
| **Bridging allo-HSCT** | | | |
| Yes vs. No# | 77.9% vs. 81.2% | 0.410 | 1.432(0.609-3.371) |
| **ABL kinase region mutation** | | | |
| Positive vs. Negative# | 49.8% vs. 84.5% | ***0.000*** | ***3.665(1.983-13.670)*** |
| **Disease** | | | |
| CML-LBC vs. Ph+ ALL# | 37.5% vs. 82.6% | ***0.004*** | ***4.263(1.624-29.110)*** |

*EMD:Extramedullary diseases; ACA:additional chromosome abnormalities; #:Control group*

**Table S6:** Treatment response and MRD negative treatment response of MFC MRD positive patients

| Prior CAR T treatment | | | Post CAR T treatment | | Treatment response | | MRD negative response | |
|---|---|---|---|---|---|---|---|---|
| MFC MRD Positive Patients N=83 | With molecular positive by PCR n=68 | CML-LBC n=4 | MFC MRD negative CR | With CMR n=1 | CML-LBC n=3 | CML-LBC 5/6<br>Ph+ ALL 77/77<br>All patients 82/83 | CML-LBC n=1 | CML-LBC 2/6<br>Ph+ ALL 65/77<br>All patients 67/83 |
| | | | | Without CMR n=0 | | | | |
| | | | MFC MRD positive CR | With CMR n=2 | | | | |
| | | | Relapse | n=1 | | | | |
| | | Ph+ ALL n=64 | MFC MRD negative CR | With CMR n=53 | Ph+ ALL n=64 | | Ph+ ALL n=53 | |
| | | | | Without CMR n=0 | | | | |
| | | | MFC MRD positive CR | With CMR n=9 | | | | |
| | | | | Without CMR n=2 | | | | |
| | With molecular negative by PCR n=15 | CML-LBC n=2 | MFC MRD positive CR n=1 | | CML-LBC n=2 | | CML-LBC n=1 | |
| | | | MFC MRD negative CR n=1 | | | | | |
| | | Ph+ ALL n=13 | MFC MRD positive CR n=1 | | Ph+ ALL n=13 | | Ph+ ALL n=12 | |
| | | | MFC MRD negative CR n=12 | | | | | |

## Supplementary data 2

# METHODS

### Definitions

Complete remission (CR) was defined as less than 5% blasts in bone marrow morphology and no extramedullary disease (EMD). MRD was assessed by multiparameter flow cytometry and MRD-negative status was defined as a leukemic cell counts below the sensitivity threshold of 1 × 10−4 (0.01%) per bone marrow mononuclear cell (MNC). MRD-negative CR was defined as MRD negative status combined with CR. A complete molecular response (CMR) was defined as a reduction in *BCR::ABL1* transcripts to ≤ 0.01%.

Response to treatment was judged by morphological, cytological or molecular level. For refractory and relapse patients, response to treatment means reaching CR. For MRD positive patients, response to treatment means persistent morphological CR or reaching CMR. CRs was defined as meeting criteria for CR with hematologic recovery (ANC ≥1 × $10^9$/L; PLT ≥100 × $10^9$/L). Bridging transplantation means HSCT performed in continuous CR after CAR T therapy. Overall survival (OS) was the time from CAR T-cells infusion to the date of death for any reason or last follow-up. Leukemia-free survival (LFS) was the duration from the day of CR after CAR T-cell therapy to leukemia relapse, death or the last follow-up.

Cytokine release syndrome (CRS) was defined as “a disorder characterized by fever, tachypnea, headache, tachycardia, hypotension, rash, and/or hypoxia caused by the release of cytokines”. “Severe CRS” was defined as 3-4 degrees of CRS. Other organ toxicities were graded according to the Common Terminology Criteria for Adverse Events Version 5.0.

## CAR T-cell products

The CD19 CAR construct contains a CD19-targeted single-chain variable fragment, the costimulatory 4-1BB domain, and the CD3 zeta domain. The CD19/CD22 CAR construct contains both CD19 and CD22 targeted single-chain variable fragment, the costimulatory 4-1BB domain, and the CD3 zeta domain. CAR T cells were prepared by the Shanghai UniCAR Technology Co.,Ltd. (UCT, Shanghai, China) and all patients received the same product.

## Statistical analysis

The differences among the two groups were analyzed by Mann–Whitney U test for continuous variables and chi-squared or Fisher's exact test for categorical variables. Multivariate logistic regression was applied to analyze response and MRD negative CR prognostic factors. OS and LFS were compared using the Kaplan–Meier method. Comparisons of survival were conducted using the log-rank test.. CIR was estimated using a competing risk model, with non-relapse mortality as a competing risk factor. Multivariate analyses of prognostic factors for LFS were conducted with Cox regression. P values <0.05 were considered statistically significant. All statistical analyses were performed with SPSS version 22, GraphPad Prism version 8.3.0 and R version 4.3.0.
